# RISK REDUCTION OF HOSPITAL ADMISSIONS FOR PEOPLE WITH DEMENTIA IN SHARED-HOUSING ARRANGEMENTS: THE DEMWG STUDY

**DOI:** 10.1093/geroni/igac059.1292

**Published:** 2022-12-20

**Authors:** Karin Wolf-Ostermann, Susanne Stiefler, Annika Schmidt, Anna-Carina Friedrich, Elmar Gräßel, Carolin Donath, Jennifer Scheel, André Kratzer

**Affiliations:** University of Bremen, Bremen, Bremen, Germany; Institute of Public Health and Nursing Research, University of Bremen, Germany, Bremen, Bremen, Germany; Institute of Public Health and Nursing Research, University of Bremen, Germany, Bremen, Bremen, Germany; Institute of Public Health and Nursing Research, University of Bremen, Germany, Bremen, Bremen, Germany; Universitätsklinikum Erlangen, Department of Psychiatry and Psychotherapy, Center for Health Services Research, Erlangen, Bayern, Germany; Universitätsklinikum Erlangen, Department of Psychiatry and Psychotherapy, Center for Health Services Research, Erlangen, Bayern, Germany; Universitätsklinikum Erlangen, Department of Psychiatry and Psychotherapy, Center for Health Services Research, Erlangen, Bayern, Germany; Universitätsklinikum Erlangen, Department of Psychiatry and Psychotherapy, Center for Health Services Research, Erlangen, Bayern, Germany

## Abstract

People living with dementia (PlwD) have an increased risk of hospital admission which may result in severe consequences. The DemWG-study therefore aims to investigate whether a complex intervention in shared housing arrangements (SHA) for PlwD reduces the number of hospital admissions. Furthermore, quality of life, risk of falls, and stabilization of cognitive abilities are assessed. The intervention includes motor and cognitive training for PlwD and education of caregivers and general practitioners. The study is conducted in 88 SHA in Germany with a total of 330 PlwD and a 6 months intervention. Data are collected up to 18 months after baseline, including quantitative and qualitative data. Results from caregivers’ perspectives on the intervention designate a perceived improved social interaction, which in turn has a positive effect on living together in the SHA. Based also on the results of the ongoing quantitative assessments recommendations for better care and support will be derived.

